# Identification of nanoparticles as vesicular cargo *via* Airy scanning fluorescence microscopy and spatial statistics†

**DOI:** 10.1039/d3na00188a

**Published:** 2023-06-14

**Authors:** Christian Wimmenauer, Thomas Heinzel

**Affiliations:** a Institute of Experimental Condensed Matter Physics, Heinrich-Heine-University Universitätsstr. 1 40225 Düsseldorf Germany thomas.heinzel@uni-duesseldorf.de

## Abstract

Many biomedical applications of nanoparticles on the cellular level require a characterisation of their subcellular distribution. Depending on the nanoparticle and its preferred intracellular compartment, this may be a nontrivial task, and consequently, the available methodologies are constantly increasing. Here, we show that super-resolution microscopy in combination with spatial statistics (SMSS), comprising the pair correlation and the nearest neighbour function, is a powerful tool to identify spatial correlations between nanoparticles and moving vesicles. Furthermore, various types of motion like for example diffusive, active or Lévy flight transport can be distinguished within this concept *via* suitable statistical functions, which also contain information about the factors limiting the motion, as well as regarding characteristic length scales. The SMSS concept fills a methodological gap related to mobile intracellular nanoparticle hosts and its extension to further scenarios is straightforward. It is exemplified on MCF-7 cells after exposure to carbon nanodots, demonstrating that these particles are stored predominantly in the lysosomes.

## Abstract 2

Many biomedical applications of nanoparticles on the cellular level require a characterisation of their subcellular distribution.

## Graphical abstract

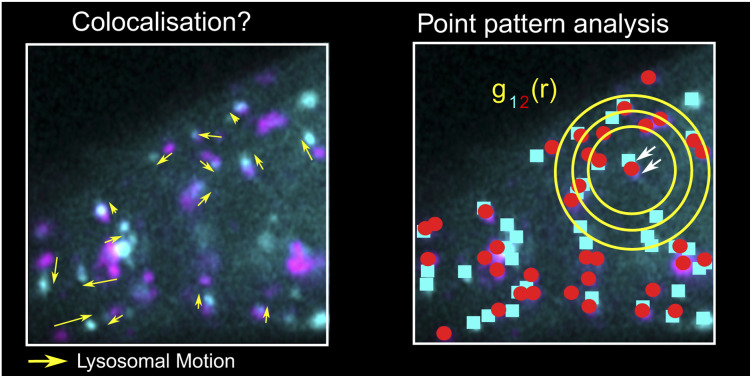

## 1 Introduction

To employ nanoparticles in biomedical applications such as drug or gene delivery, it is mandatory to determine their subcellular location after uptake. A common approach relies on using fluorescent or fluorescence-marked particles in combination with staining the candidate organelles, after which the system is studied by fluorescence imaging and subsequent co-localization analysis. This powerful concept has provided many highly relevant results, like the clarification of viral uptake mechanisms^1,2^ or the characterisation of a variety of potential nanoparticle systems for drug delivery and photodynamic therapy.^3–5^ However, the technique experiences also several limitations. One of these arises in a typical scenario, where the two fluorescence channels have to be detected with the same sensor, thus requiring sequential sampling of the two images for each channel and subsequent data analysis. In this case, the measured spatial correlation can be disturbed by the motion of the nanoparticles and vesicles during the single image data acquisition time. *Object based colocalization methods* have been established which relate spatially the structures in both channels after a segmentation step. Prominent methods from this field include Voronoi tessellation,^6^ trajectory correlation^7^ and overlap analysis.^8^ More recently, methods from spatial statistics found their way into this field, with the best known example being the bivariate version of Ripley's *K*-function.^9,10^ Further numerical instruments such as the pair correlation function^11^ (PCF), the estimation of interaction probabilities based on point patterns^10,12,13^ or the nearest neighbour function (NNF)^8^ have also been successfully applied to co-localization problems. In some cases, however, these powerful methods also experience limitations, in particular for vesicles that move significantly on the time scale set by the microscopy scans and regarding the spatial resolution limits by conventional confocal microscopy.

In the present work, we show that these problems can be reduced or avoided altogether, respectively, by a combination of super-resolution microscopy, here in the form of Airy scanning fluorescence microscopy, with a subsequent spatial statistics analysis which we denote by *SMSS* in the following. Airy scanning microscopy yields a higher resolution and superior signal-to-noise ratio compared to conventional confocal imaging, as required for vesicles with sizes below ≈500 nm. We apply the PCF and the NNF to the measured data and create envelopes representing 95%-confidence intervals. They are based on Monte-Carlo-simulations of complete spatial randomness (CSR) and a model for transport with heavily tailed jump distances. It is shown that these envelopes are of relevance for hypothesis testing and for proving consistency of the measured data with an underlying assumption.^14,15^

As a topical model system, MCF-7 cells incubated with ultra-small carbon nanodots (CNDs) are used. Even though the family of CNDs is relatively diverse,^16^ common features such as good solubility in water,^17,18^ low toxicity^19,20^ and accessible functionalization protocols^21,22^ put them in the focus for biomedical applications. Proof of principle experiments for drug delivery,^23–25^ photosensitizing,^26,27^ deep tissue imaging^28,29^ and intracellular sensing^18,29^ have been reported. Their intracellular storage in different target organelles depends on the composition, surface functional groups and periphery of the particle.^30^ While several groups have identified the vesicles of the endolysosomal system as primary location,^31,32^ they appear to be stored in the Golgi apparatus,^33^ mitochondria^34^ or in the nuclei^35^ in other experiments. We have designed a study of the colocalization of CNDs with lysosomes. The computed statistical functions are compared with model envelopes and are discussed in the context of a positive control (double labelled lysosomes) and a negative control (labelled lysosomes and Golgi apparatus).

The same detector is used for both fluorescence channels to be compared, which thus have to be acquired by sequential scanning. Sequential scanning is favored in some colocalization problems, since it minimizes the bleed through from a fluorophore with a broad emission spectrum into the other channel, which is detrimental to colocalization analysis. Especially for nanoparticles with intrinsic fluorescence properties it may be, therefore, hard to optimize for a simultaneous acquisition. Furthermore, limitations in the available instrumentation may pose a problem since for sophisticated methods like Airy scanning or STED simultaneous acquisition of multiple fluorophores, while possible, sets high requirements on the setup. Since the acquisition of an image takes up to 2 s, intensity correlation based colocalization analysis fails due to the vesicular motion by a distance comparable to, or even larger than, their size within the acquisition time of one frame, see Fig. 3 (zoom-in, second row). Therefore, in this typical scenario, an object based analysis method needs to be applied.

## 2 Materials and methods

### 2.1 Carbon nanodots

#### 2.1.1 Synthesis

CNDs were prepared *via* a slightly modified version of the bottom up synthesis method proposed by Qu *et al.*^17^ as described in detail elsewhere.^19^ 0.21 g anhydrous citric acid (Alfa Aesar) and 0.34 g diethylenetriamine (Merck) were mixed and treated in a closed microwave reaction chamber (CEM Discover) at 180 °C for 150 s under continuous stirring. The resulting product was dissolved in DI water, placed in a 100–500 Da dialysis tube (Float-A-Lyser) and dialysed against 2 L of DI water for 48 hours. The water was exchanged three times during the process. The dialysis product was lyophilized to obtain the dry mass and redissolved in phosphate-buffered saline (PBS, Gibco) and sterile filtered (pore size 0.22 μm, Satorius).

#### 2.1.2 Characterisation

The as prepared CNDs were characterized *via* Raman spectroscopy, transmission electron microscopy (TEM), X-ray photoelectron spectroscopy (XPS), CHN elemental analysis, fluorescence spectroscopy and atomic force microscopy (AFM) in previous work.^19^ This section will briefly summarize the already published findings. In Raman spectroscopy a pronounced G-band around 1596 cm^−1^ corresponding to graphitic sp^2^-carbon as well as a D-, D1-, D2- and D3-band at (1375, 1195, 1264 and 1412 cm^−1^) corresponding to different sp^3^-carbon containing groups were found. In TEM measurements on an amorphous carbon substrate nanoparticles with an average diameter of 3.3 nm and a lattice with an hexagonal symmetry and a lattice constant of 0.223 nm were found. Taking lattice strain due to impurities and the limited size of the particles into account, this value is in good agreement with the lattice constant of bulk graphene (0.246 nm). In CHN elemental analysis the mass fractions were determined to be 40% carbon, 8% hydrogen and 19% nitrogen. Attributing the remaining mass fractions to oxygen is consistent with the results from the XPS measurement. From AFM measurements on silicon oxide the height was determined to range between 1 nm and 2 nm, which is in good agreement with the height of two to three layers of graphene on this kind of substrate.

The absorbance spectrum shows two pronounced peaks in the UV-region, one around 230 nm commonly attributed to the π–π*-transition of the graphitic carbon domains and one around 350 nm which is attributed to the n–π*-transition of C=O moieties.^17^ The employed CNDs display their strongest emission around 460 nm, if excited at 360 nm. A quantum yield of 23% was obtained for these particles.

### 2.2 Cell experiments

#### 2.2.1 Cell culture

MCF-7 cells, a breast cancer cell line from an invasive breast ductal carcinoma, were cultivated in medium consisting of RPMI 1640, 10% fetal bovine serum and 1% penicillin streptomycin solution. The cells were maintained at 37 °C and 5% CO_2_ in a Heracell 150i (Thermofisher Scientific™) incubator in a 75 cm^2^ cell culture flask. The culture was split every three to four days using a trypsin/EDTA solution (Sigma-Aldrich).

#### 2.2.2 Transfection and incubation with CNDs

MCF-7 cells are seeded out at a density of (10 000–15 000) cells per well in an 8 well μ-slide (no. 1.5 polymer coverslip, tissue culture treated, Ibidi™) 48 hours prior to imaging. CNDs are added to respective wells to yield a concentration of 500 μg mL^−1^. The cells in the respective chambers were transfected with reagents from the CellLight™ BacMam 2.0 product series (Invitrogen™) 24 hours prior to imaging. To label the lysosomes CellLight™ Lysosomes-RFP and CellLight™ Lysosomes-GFP was used yielding MCF-7 cells expressing a fusion protein of LAMP1 and the respective fluorescent protein. To label the Golgi apparatus CellLight™ Golgi-GFP was used yielding MCF-7 cells expressing a fusion protein of the human Golgi resident enzyme *N*-acetylgalactosaminyltransferase and GFP. One hour before imaging the samples were washed with PBS and the medium was exchanged with fresh full medium. Between the preparation steps the sample was kept in the incubator at 37 °C and 5% CO_2_.

#### 2.2.3 Microscopy

The as treated specimen were imaged using a Zeiss LSM 880 Airyscan confocal microscope with an 63× oil objective (Planachromat, NA 1.4) at 37 °C. Owing to the employed 32-channel gallium arsenide phosphide photomultiplier tube (GaAsP-PMT) area detector with each detector element collecting a full confocal image the Zeiss LSM 880 Airyscan has an improved signal-to-noise ratio compared to conventional confocal microscopy. It can be run in *superresolution mode* with a lateral resolution of down to 140 nm or in *fast mode* collecting multiple line scans in parallel resulting in a significantly reduced scan time.^36,37^ The images were acquired in *superresolution mode* and time series in *fast mode*. To image the CNDs, a 405 nm laser diode was used for excitation. For GFP excitation, an argon laser with a wavelength of 488 nm was used for excitation and RFP was excited by a 561 nm diode pumped solid state laser was used for excitation. In all measurements channels were acquired frame wise.

### 2.3 Data analysis

**Fig. 1:**
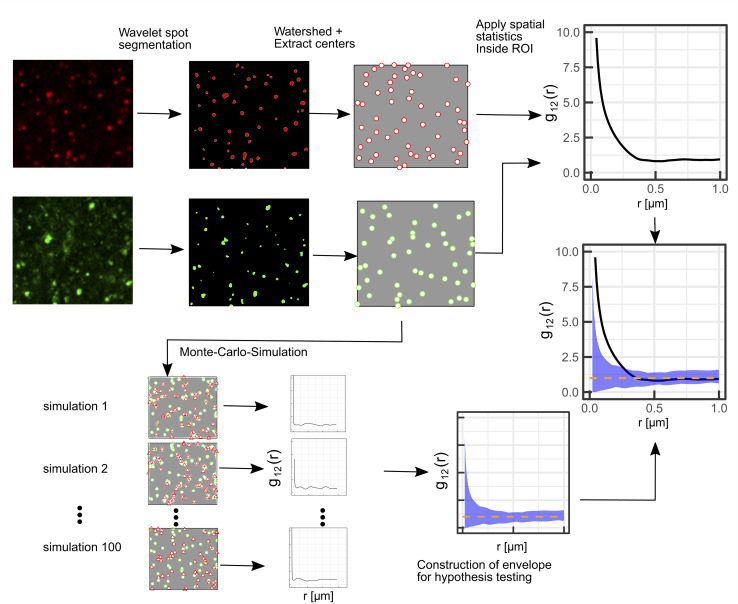
Illustration of the proposed protocol comprising Airy scanning fluorescence microscopy in the superresolution mode in combination with spatial statistics (SMSS). First, a wavelet segmentation is applied to extract the spots in each channel, then the watershed method is applied and the centers of the spots are extracted. Bivariate spatial statistics is applied to yield the summary function, *i.e. g*_12_(*r*) in this illustration. To test the distribution against a random distribution of lysosomes in the red channel, the green points are used as a starting point for a Monte-Carlo-simulation. From the Monte-Carlo-simulations for spatial randomness (red triangles in the frames to the lower left) an envelope is constructed that encloses the 95 least extreme values of the summary functions from the simulations (blue shaded area). The dashed orange line denotes the mean value of the simulated summary functions for spatial randomness.

A schematic representation of the SMSS protocol is shown in Fig. 1.

Wavelet segmentation is performed *via* the implementation of the open image analysis platform Icy.^38^ The watershed method is then applied to the binary image, and the point pattern is extracted with the analyze particles function implemented in Fiji.^39^

#### 2.3.1 Bivariate spatial statistics

Programs to perform the spatial point pattern analysis make extensive use of functions from the *spatstat* package^40^ for the R programming language.

The bivariate *G*-function1
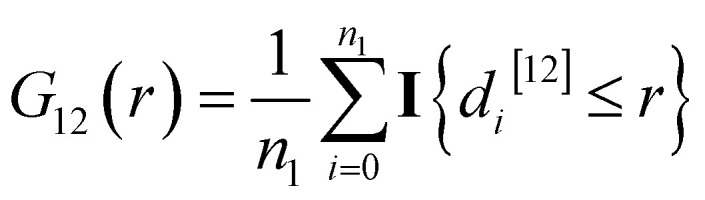
measures the number of spots with a nearest neighbour distance below *r*, and *d*_*i*_^[12]^ denotes the nearest neighbor distance between spot *i* of type 1 and the type 2 spots. Here, **I**{*Y*} is the indicator function that takes the value of one if the statement *Y* is true and gets zero if *Y* is false.

The bivariate pair correlation function is related to the probability of finding a spot of type 1 a given distance *r* from a spot of type 2. It is defined *via* the derivative of the bivariate *K*-function2
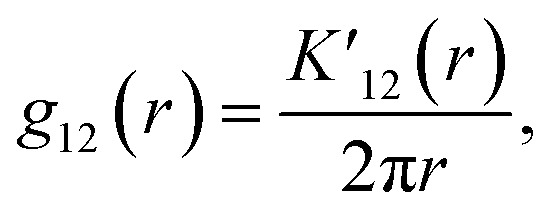
with the bivariate *K*-function3
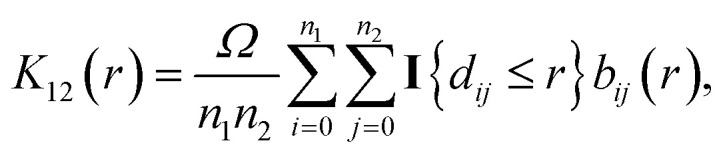
which measures the number of type 2 spots inside of a circle with radius *r* and around a type 1 spot the area of the region of interest (ROI) is denoted by *Ω* and the number of spots is denoted by *n*. The expression *b*_*ij*_(*r*) corrects for edge effects. In our particular case, the translation correction^41^ is applied. The manually estimated borders of the cell from the autofluorescence were selected as ROI. The nucleus was excluded from the ROI to prevent artificial inflation of the summary functions (images of the ROIs are available in the ESI†). Model calculations of *G*_12_(*r*) and *g*_12_(*r*) for clustered and for periodically arranged spots are reproduced in the ESI.† Since in contrast to *g*_12_(*r*), *G*_12_(*r*) neglects points beyond the nearest neighbor but has a more intuitive interpretation, giving the share of points that found their nearest neighbor up to a distance of *r*, these two summary functions are complementary and we discuss both of them below.

For the interpretation, the spatial statistical functions as obtained from the measured data are compared to *reference envelopes*. To construct them, an ensemble of *N* = 100 simulated point patterns is generated under a model assumption. For these simulated point patterns an ensemble of summary functions is calculated, capturing the behaviour of the model. For a significance level *α* = 0.05 the 5% that deviate the most from the mean are excluded from the ensemble of simulated summary functions at each distance *r*. The minimum and maximum of the remaining points describe the borders of the envelope. If the respective summary function of the measured data lies outside of the envelope inside of a certain interval in *r*, the *p*-value is grater than the significance level (*p* > *α*) and the underlying model (null hypothesis) is rejected in this interval. The first tested model was a uniform random distribution inside the ROI of the type 2 spots (red channel), while the type 1 spots had the same distribution as the measured data. The underlying null hypothesis is that the positions of the type 2 spots are independent of the type 1 spots. In the second tested model the positions of the type 1 spots are again the same as for the measured data. To test the null hypothesis of transport with a lognormal distribution of jump distances the simulated type 2 spots were generated by shifting each of the type 1 spots by a vector 
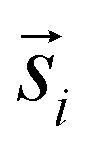
. Length 
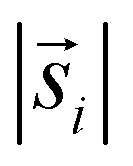
 was sampled from a lognormal distribution that was fitted to the jump distance distribution of the measured data and the angle *θ* that describes the direction was sampled form a uniform distribution. If a simulated point of type 2 was generated outside of the ROI a new vector 
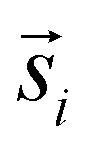
 was generated. To ensure that the number of type 2 points in every simulation (*n*_s_) corresponds to the number of type 2 points in the measurement (*n*_m_), spots in random locations were added (if *n*_m_ > *n*_s_) or the set of simulated points was sub-sampled to match *n*_m_ (if *n*_m_ < *n*_s_).

#### 2.3.2 Single particle tracking and trajectory analysis

To determine the particle trajectories from the microscopy data the *TrackMate* plugin^42^ for the open source image analysis platform *Fiji*^39^ is used. The particle positions were detected with the *Laplacian of Gaussians* detector (*d* = 0.8 μm) and detected objects were filtered afterwards based on mean intensity and quality. With the *Simple Linear Assignment Problem Tracker*^43^ with a linking distance of 1 μm, a maximum gap of 2 frames and a gap closing distance of 2 μm, the lysosomal trajectories are constructed. Merging or splitting events were disregarded. Trajectories with less than ten time points or more than five gaps were excluded.

## 3 Results and discussion

### 3.1 Single particle tracking of lysosomes

Lysosomes are actively transported *via* kinesin and dynein along the microtubuli,^46^ which manifests itself in short bursts of directed motion. In between these intervals of active transport, lysosomes show (sub-) diffusive behaviour, which has been attributed to lingering at the intersections of microtubuli.^47^

**Fig. 2:**
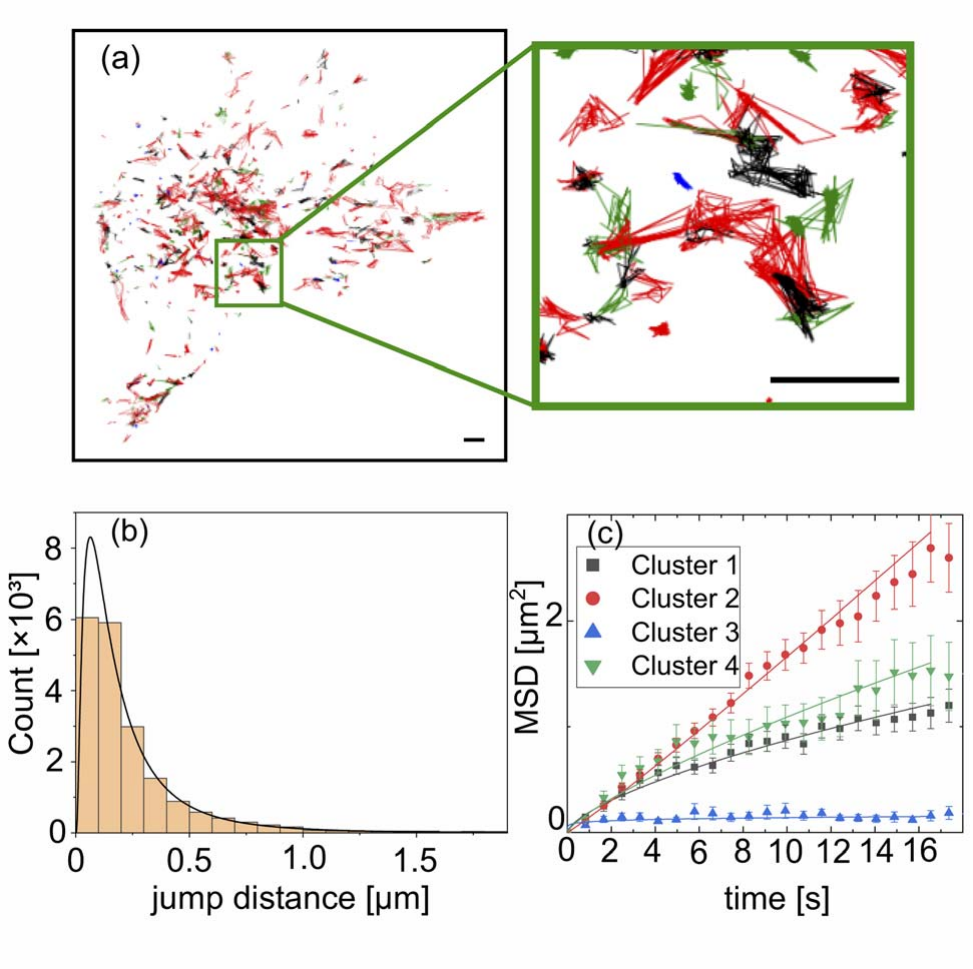
Single particle tracking of lysosomes. (a) Extracted single particle trajectories colored according to the identified cluster *via* cluster analysis with *t-distributed stochastic neighbor embedding* (tSNE)^44^ and *density-based spatial clustering of applications with noise* (DBSCAN).^45^ The size of the scale bar amounts to 5 μm. (b) A lognormal distribution was fitted to the frame to frame jump distance for all trajectories. (c) Three different subtypes of lysosomal motion, namely immobile, subdiffusive and diffusive, were identified for the four clusters by inspection of their MSD scaling.

This bimodal motion resembles a Lévy walk.^48–50^ To construct envelopes for the assumption of transport, knowledge about the jump distance distribution is necessary. To obtain insight in the underlying transport dynamics, single particle tracking was performed on a time series measurement over 100 time points with an acquisition time of 827 ms per frame. After excluding trajectories shorter than 8.3 s, 763 trajectories remain, which form the input of the analysis. Typical trajectories are reproduced in Fig. 2(a).

The distribution of frame-to-frame jump distances is heavy tailed and can be described reasonably well by a log-normal distribution with *σ* = 0.94 and *μ* = −1.87, see Fig. 2(b), in agreement with recent results.^50^ Following a similar workflow to the method presented by Pinholt *et al.*,^51^ the trajectories are classified prior to an analysis of their time dependent ensemble mean square displacement (MSD) as follows. Eight descriptive features, *i.e.*, the mean, standard deviation, skewness and kurtosis of the jump distance distribution, as well as the straightness, sinuosity, average MSD and turn angle correlation^52^ are calculated for each trajectory, providing a space for classification *via* the density based clustering algorithm *density-based spatial clustering of applications with noise* (DBSCAN).^45^ A non-linear dimensionality reduction *via t-distributed stochastic neighbor embedding* (tSNE)^44^ was applied prior to the clustering. From the six emerging clusters, see the ESI† for detailed information, two consisted of very few points and where therefore excluded from the subsequent MSD analysis. Fig. 2(c) shows the resulting averaged MSD curves for the three clearly distinguishable extracted states (full curves in ESI†). Fitting the data to the standard expression MSD = 4*Dt*^*α*^ gives the fit parameters as summarized in displayed in Table 1. Class 1 (cluster 2, red), which forms by far the largest group, is diffusive, *i.e.*, *α* ≈ 1. The trajectories of class 2 (cluster 3, blue) are immobile to a good approximation, while class 3 (cluster 1, black and cluster 4, green) is subdiffusive.

**Table 1:** Results of the power law fit to the MSD functions displayed in Fig. 2(d). Displayed are the type of motion, the effective diffusion constant *D* and the exponent *α* from the expression MSD = 4*Dt*^*α*^, the number of trajectories in the cluster *N* and the coefficient of determination *R*^2^

Cluster	Type	*D* [μm^2^ s^−1^]	*α*	*N*	*R* ^2^
1	Subdiffusive	0.048 ± 0.003	0.66 ± 0.03	147	0.98
2	Diffusive	0.036 ± 0.002	1.06 ± 0.019	466	0.99
3	Immobile	0.025 ± 0.003	0.14 ± 0.06	33	0.30
4	Subdiffusive	0.047 ± 0.004	0.77 ± 0.05	108	0.95

Typical sample trajectories of the three classes are shown in Fig. 2(a). Class 1 trajectories correspond to lysosomes travelling predominantly along filaments without changes of the direction by large angles. The lysosomes with class 2 trajectories are essentially captured at some structure, for example a filament junction. Lysosomes travelling along the filaments with frequent large-angle changes of the path form class 3. The values for the diffusion constant are within a reasonable range compared to the literature.^50,53^ It should be noted that this classification treats the trajectories globally, in contrast to segmentation of the trajectories into different diffusive states, in which bursts of directed motion appear on smaller time scales below 10 s.^50^ A finer temporal resolution in combination with a more sophisticated segmentation algorithm may provide more insight into the lysosomal movement patterns on a single particle level, but is beyond the scope of this work. These studies show that a significant fraction of the lysosomes move farther than their size within the capture time of a picture frame, which suggests them a suitable test bed for the SMSS analysis.

### 3.2 Control experiments

**Fig. 3:**
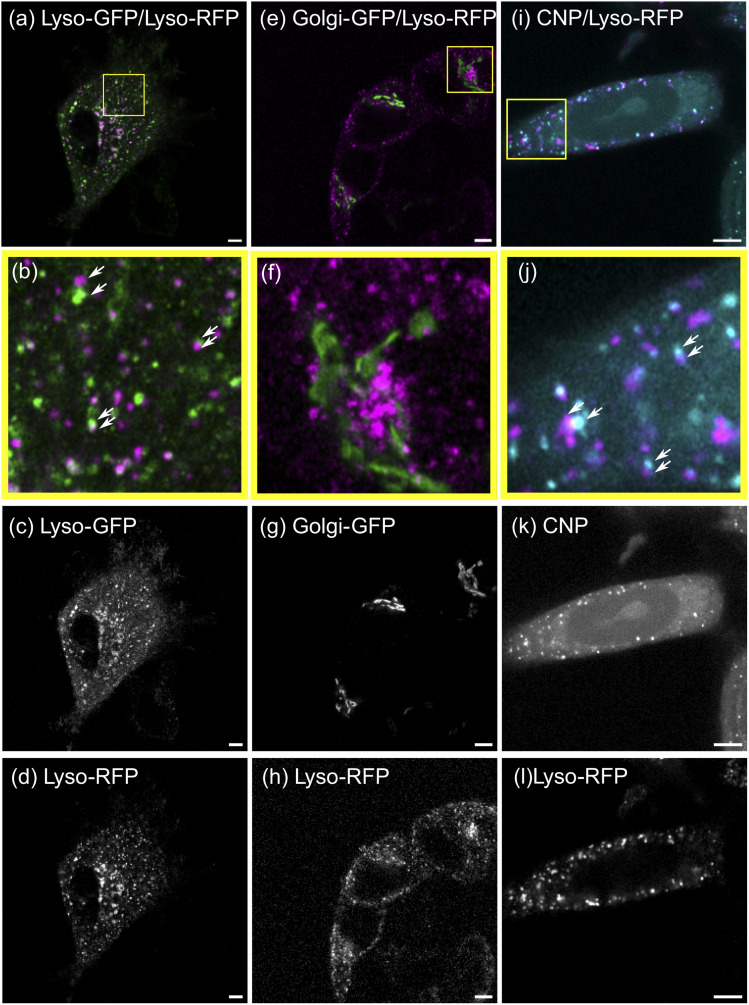
Microscopy images of MCF-7 cells with RFP- and GFP-labeled lysosomes (left column, (a)–(d)), RFP-labeled lysosomes and a GFP-labeled Golgi apparatus (middle column, (e)–(h)) and RFP-labeled lysosomes post CNP incubation (right column, (i)–(l)). The top row (a, e, i) displays the overlay of both channels, while the individual channels are shown in the lowermost two rows (c, g, k and d, h, l, respectively). The second row (b, f, j) shows a zoom in on the yellow bordered region in the composite images. The white arrows indicate spots in different channels of same size and shape that are in close proximity. These indicated spots supposedly correspond to the same object that is shifted due to movement between the acquisitions of both channels. The length of all scale bars amounts to 5 μm.

**Fig. 4:**
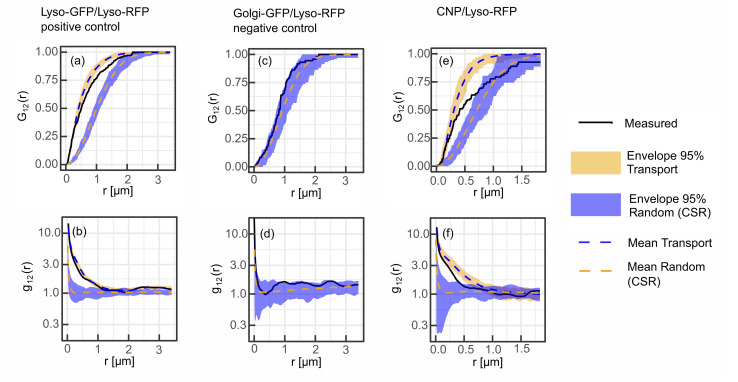
Results of point pattern analysis. Bivariate spatial statistics was applied to all three conditions namely the positive control with lysosomes labelled in both channels (left column, (a) and (b)), the negative control displaying lysosomal markers in channel 1 and the Golgi apparatus in channel 2 (middle column, (c) and (d)) and the condition with lysosomal staining visible in channel 1 and the distribution of the CNDs in channel 2 (right column, (e) and (f)). The set of summary functions comprises of the nearest neighbor function *G*_12_(*r*) (top row, (a), (c) and (e)) and the pair correlation function *g*_12_(*r*) (bottom row, (b), (d) and (f)). The summary functions are displayed as solid black lines, while the envelopes for uniform randomization of the lysosomal channel and a model for transport with a heavy tailed jump distance distribution are displayed in blue and orange respectively.

To validate the SMSS protocol, we perform two control experiments and determine their corresponding reference envelopes. In the positive control experiment, MCF-7 cells are transfected to express a fusion protein of the lysosome resident protein LAMP1 and RFP as well as a fusion protein of LAMP1 and GFP. Fig. 3(a)–(d) reproduce characteristic fluorescence microscope images. Since both channels capture the location of the same subcellular structure the employed method is expected to yield maximum colocalisation under the present experimental conditions. The two bivariate functions *G*_12_(*r*) and *g*_12_(*r*) of the experimental data should therefore reside inside the reference envelope for the transport model, *i.e.* spatial correlation. As can be seen in Fig. 4(a) and (b), respectively, the measured bivariate functions lie inside the confidence intervals for *r* ≤ 500 nm, deviate slightly from them for larger distances, but remain close up to the largest distances studied, *i.e.* 3.3 μm. The reference envelope for spatial independence, on the other hand, is distinctly separated from the experimental data, showing some overlap only at large distances above *r* = 1.8 μm for *G*_12_(*r*), and in the range *r* = 1.2 μm for *g*_12_(*r*). As an interpretation example, we evaluate that therefore, for distances *r* < 1.2 μm, the null hypothesis of the spatial randomness can be rejected with *p* < 0.05. From *G*_12_(*r* = 1.2 μm) ≈ 0.8, on the other hand, we can infer that 80% of points in the green channel have a nearest neighbor distance of less than 1.2 μm to points in the red channel. This positive control experiment shows how a close to perfect cross-correlation will look like in our experiments. We explain the deviations between the measured trace and the reference envelope for correlation at larger distances with the three-dimensional character of the cell. As soon as lysosomes move into or out of the focal plane, the correlation is blurred. This will set in at the focal length of the microscope, which in our case equals approximately 500 nm. Furthermore, the treatment of points exiting the edge of the ROI in the simulation may also introduce a bias. If a simulated point is generated outside of the ROI, a new point is generated until it lies inside of the ROI. This treatment may introduce a bias that favours small jump distances for points close to the cellar border. Nonetheless, reasonable agreement of the measured data with the transport model persists.

For the negative control experiment, the cells were transfected to express a LAMP1/RFP fusion protein labeling the lysosomes, but this time co-labeled with a fusion protein for an enzyme resident in the Golgi, namely *N*-acetylgalactosaminyltransferase with GFP, see Fig. 3(e)–(h) for fluorescence microscopy images. The labeled organelles are spatially disjunct and correlate only in the sense that their locations mutually exclude each other. Therefore, the bivariate functions are expected to reside inside the reference envelope for spatial independence. As can be seen in Fig. 4(c) and (d), both *G*_12_(*r*) and *g*_12_(*r*) lie inside the reference envelope, *i.e.*, the null hypothesis can not be rejected. It can therefore be inferred that the spatial spot pattern for the negative control is indistinguishable from an independent distribution for the two spot types.

### 3.3 Spatial correlation between CNDs and lysosomes

With the control experiments at hand, we apply the SMSS protocol to quantify the subcellular localisation of the CNDs. MCF-7 cells are transfected to express the LAMP1/RFP fusion protein and are afterwards incubated with CNDs for 48 h, leading to typical bicolor fluorescence patterns as shown in Fig. 3(i)–(l). The resulting bivariate functions are reproduced in Fig. 4(e) and (f) together with the reference envelopes. For distances *r* < 0.5 μm, both spatial statistical functions lie outside the reference envelope for independent motion. Thus, the hypothesis of the lysosomal distribution being independent of the CND distribution can be rejected with *p* < 0.05. Rather, *G*_12_(*r*) and *g*_12_(*r*) are found inside the envelope functions for correlated motion for small distances. From *G*_12_(*r* = 0.5 μm) > 0.5, see Fig. 4(e), we conclude that more than 50% of the nearest neighbor distances between CNDs and lysosomes are smaller than 0.5 μm. At larger distances, *G*_12_ runs outside this reference envelope but, as we have seen during the study of the correlated reference envelope, this is the case even for perfect correlation and can be explained by lysosomes CNDs entering or leaving the focal plane within the period set by the single picture acquisition time. These results show that with a significance level of 0.05, more than 50% of the fluorescent CNDs are captured in the lysosomes in our experiments, which therefore form the dominant host for the CNDs.

### 3.4 Generalizability and critical evaluation of the method

The proposed SMSS protocol can be applied to explore the subcellular distribution of a wide variety of nanoparticles, since it requires simply that the nanoparticle possess intrinsic fluorescence and a moderate photostability, in order not to bleach out on the time scale of the acquisition. It may be furthermore useful for nanoparticles with a wide emission spectrum, which benefit from sequential scanning in colocalization experiments to minimize bleed through. The presented workflow is modular and may be adapted to a given problem. First of all, the presented segmentation pipeline may not be appropriate in all situations. Wavelet based segmentation was used since it is known that this approach is well-suited to extract small spots even for varying levels of intensity. For more specialized problems, other segmentation pipelines^54,55^ may be employed. Second, the transport characteristic that is used to model the envelope for transport has to be evaluated on a case-by-case basis. While a log-normal distribution of jump distances is well-suited for the presented problem, this cannot be applied to all transport process in a cellular context. In the case of normal diffusion, for example, a Gaussian distribution of the displacement in *x*- and *y*-direction would be more appropriate. We illustrate the analysis of diffusive objects on synthetic data found in the ESI.† Directed motion with a negligible diffusive component, for example, has to be treated differently again.

Furthermore, application of the SMSS concept requires sufficiently dilute objects in the following sense. The mean spacing between the objects to be traced sets the length scale of the objects' motion distance in between two consecutive recording times: if objects move more than the mean spacing during this time interval, complete spatial randomness (CSR) and the underlying transport process can no longer be distinguished, leading to a significant overlap of the respective envelopes. This is illustrated by synthetic data in the ESI† showing the analysis of points subject to a radial drift. At high object densities, the directed motion of the ensemble becomes indistinguishable from CSR. Finally, optical aberrations may interfere with the spatial statistics analysis. Chromatic aberrations in particular may present a problem since they distort the location of the objects in both fluorescence channels differently. As for all fluorescence colocalization experiments it is essential to minimize this effect by centering the region of interest on the optical axis of the imaging system and using achromatic objectives. If a significant amount of chromatic aberration is present, the image would be subject to stretching that increases from the center to the periphery.

## 4 Conclusions

It has been demonstrated that Airy scanning fluorescence microscope in combination with spatial statistics functions and reference envelopes provides a powerful tool to quantify correlated motion of nanoparticles and/or vesicles on a subcellular level. It is particularly suited for setups where locations measured in subsequent pictures may be correlated, which may be for instance necessary to minimize bleed through when fluorophores with a broad emission spectrum must be used. The reference envelopes corresponding to the hypotheses to be tested (here: uncorrelated motion and motion with a log-normal distribution) enable the specification of significance levels according to the requirements (set to the frequently used value of 0.05 in our work). This rather broadly applicable scheme, with selectable targets for labelling as well as several established spatial statistical functions, has been exemplified by a study the spatial colocalization of carbon nanodots in lysosomes. The results do not exclude that a fraction of the CNDs reside inside the Golgi apparatus or the nucleoli, for example. This would require additional experiments along the lines discussed above. However, it can be concluded that the major fraction of the CNDs is localized inside mobile lysosomes with a significance level of 0.05. The experienced limitations of the SMSS protocol in its present form have been identified being manly due to motion of the detected fluorescent units into or out of the focal length of the microscope. Therefore, confinement of the system under study to a plane with a thickness of about 500 nm appears to be one promising future way to improve the presented protocol.

## Conflicts of interest

There are no conflicts to declare.

## Supplementary Material

NA-005-D3NA00188A-s001
