# Supplementary material for: Identification of nanoparticles as vesicular cargo via Airy scanning fluorescence microscopy and spatial statistics
Source: Nanoscale Adv. 2023 Jun 14;5(13):3512–20. doi: 10.1039/d3na00188a (PMC10295176; doi:10.1039/d3na00188a)
Supplement: NA-005-D3NA00188A-s001 [file NA-005-D3NA00188A-s001.pdf]

Electronic Supplementary Material (ESI) for Nanoscale Advances.
This journal is © The Royal Society of Chemistry 2023

Supplementary Information: Identification of nanoparticles as vesicular cargo via Airy scanning fluorescence microscopy and spatial statistics

Christian Wimmenauer[a] and Thomas Heinzel*[a]

[a]*Institute of Experimental Condensed Matter Physics, Heinrich-Heine-University, Universitätsstr. 1, 40225 Düsseldorf, Germany, E-Mail: thomas.heinzel@uni-duesseldorf.de*

1 Details Spatial Statistics

1.1 Spatial statistics summary functions

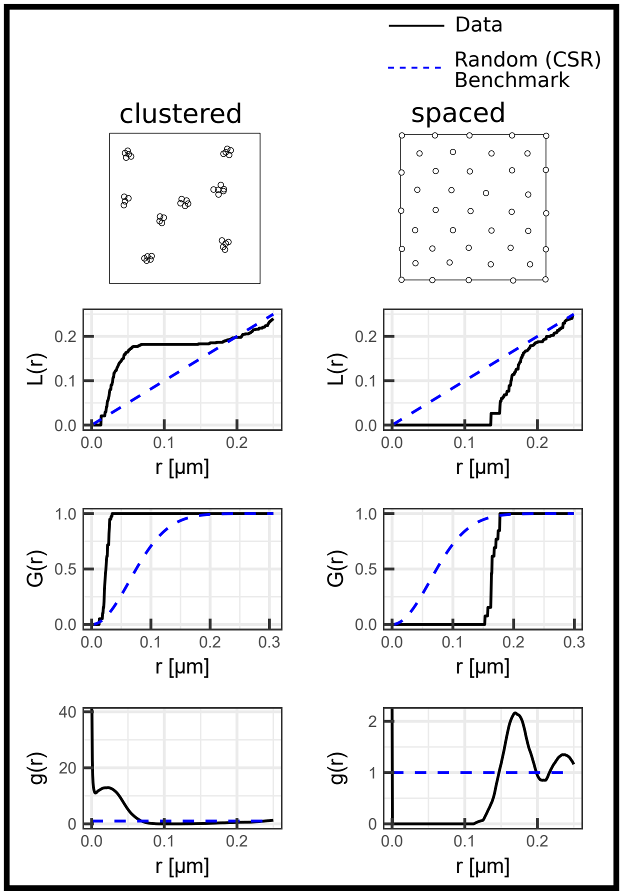

Figure 1: Univariate summary functions of a manually generated clustered (left) and a spaced (right) point pattern to illustrate the respective behaviour in these cases.

1.2 Monte Carlo Simulations

1.2.1 Complete Spatial Randomization of a single channel

To generate point patterns that display complete spatial randomness in one of the color channels the points in color channel 2 are sampled from a continuous uniform distribution inside the region of interest (ROI), while the positions of the points in channel 1 in the generated pattern match the positions in the measured data. The number of generated random points was chosen to match the number of points from the measurement.

1.2.2 Transport Simulation

First a lognormal distribution

$$lnorm_{\mu,\sigma}(x) = \frac{1}{\sqrt{2\pi}\sigma x} \exp\left[\frac{(\log(x)-\mu)^2}{2\sigma^2}\right]$$

is fitted to the histogram of nearest neighbour distances between the first and second color channel points in the point pattern, to estimate reasonable parameters for the mean μ and standard deviation σ on the logarithmic scale. The positions of the channel 1 points in the simulation are unaltered compared to the measurement $\vec{r}'_{1,i} = \vec{r}_{1,i}$. The positions of the channel 2 points in the simulation $\vec{r}'_{2,i}$ are calculated by shifting the positions of the channel 1 points $\vec{r}_{1,i}$ by $\Delta\vec{r}_{\mu,\sigma,i}$.

$$\vec{r}'_{2,i} = \Delta\vec{r}_{\mu,\sigma,i} + \vec{r}_{1,i}$$

The shift vector $\Delta\vec{r}_{\mu,\sigma,i}$ is randomly sampled

$$\Delta\vec{r}_{\mu,\sigma,i} = \rho_{\mu,\sigma} \begin{pmatrix} \cos(\theta) \\ \sin(\theta) \end{pmatrix}$$

, where $\rho_{\mu,\sigma}$ is sampled from $lnorm_{\mu,\sigma}(x)$ and $\theta \in [0, 2\pi]$ is sampled from a continuous uniform distribution. If the resulting $\vec{r}'_{2,i}$ lies outside of the defined ROI a new shift vector $\Delta\vec{r}_{\mu,\sigma,i}$ is sampled until the resulting $\vec{r}'_{2,i}$ lies inside of the ROI. This process is repeated for every channel 1 point $\vec{r}_{1,i}$.

The number of generated points is subsequently altered to ensure that the number of generated points in channel 2 is equal to the number of measured points in channel 2. If the number of points in channel 2 in the measurement n_m is greater than the number points in channel 2 in the simulation n_s ($n_m > n_s$), a total of $n_m - n_s$ channel 2 points sampled from a continuous uniform distribution in the ROI are added to the simulated point pattern in channel 2. If on the other hand $n_m < n_s$, a total of $n_s - n_m$ randomly selected channel 2 points are removed from the simulated point pattern.

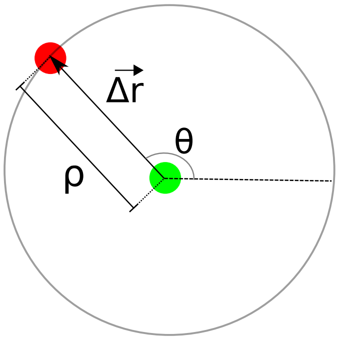

Figure 2: Construction of the shift vector Δr from the randomly sampled variables ρ and θ

2 Colocalisation: Supporting Data

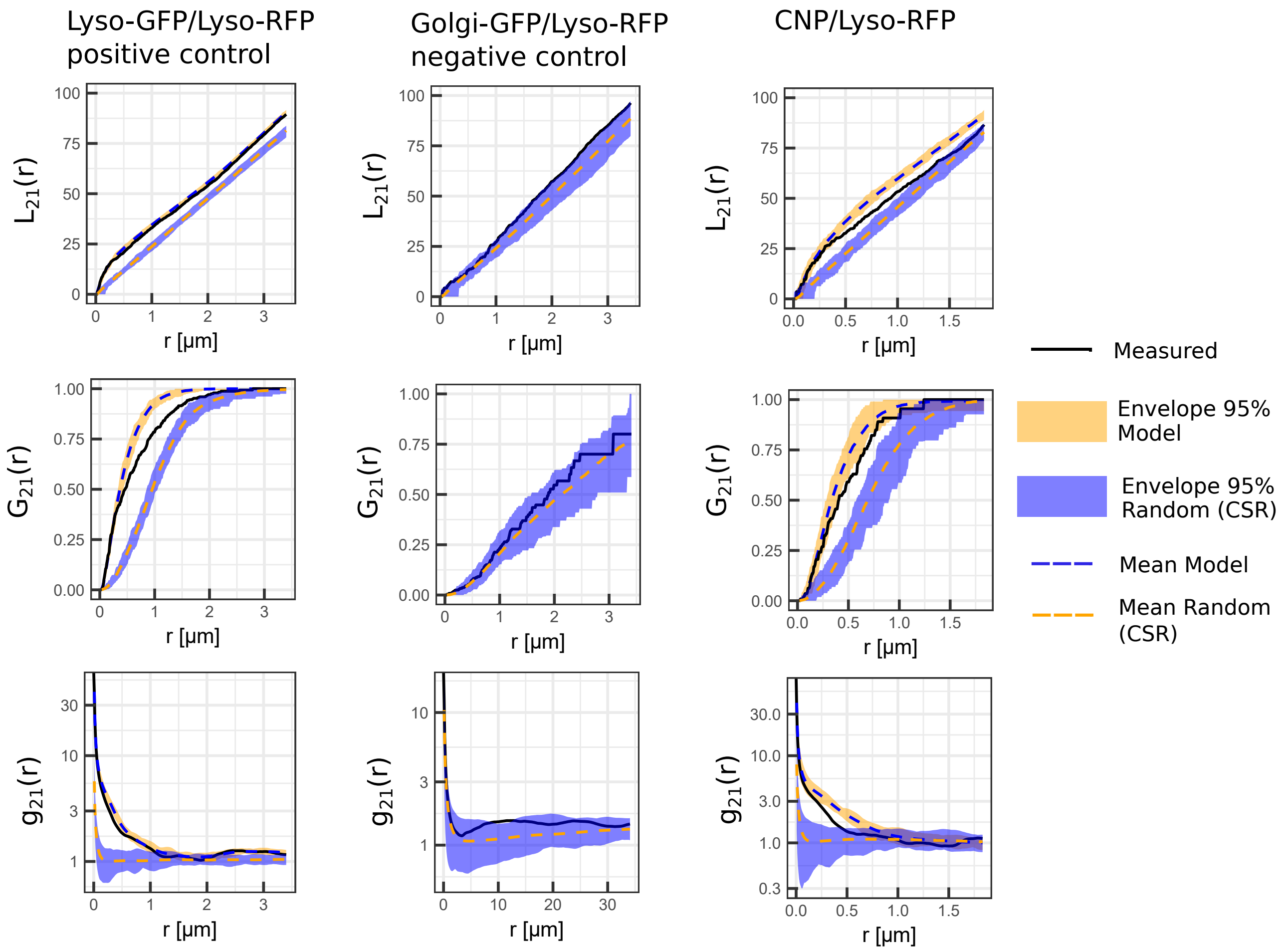

Figure 3: Complementary summary functions to the panel in the main article. Here the color channels 1 and 2 have been exchanged in the calculation of the bivariate summary functions (e.g. $g_{21}(r)$ is shown instead of $g_{12}(r)$). It becomes apparent, that the character of the summary functions persists. Furthermore, the L-Function is shown in this panel, which is in large parts redundant to the pair correlation function and is thus not dicussed in the main article.

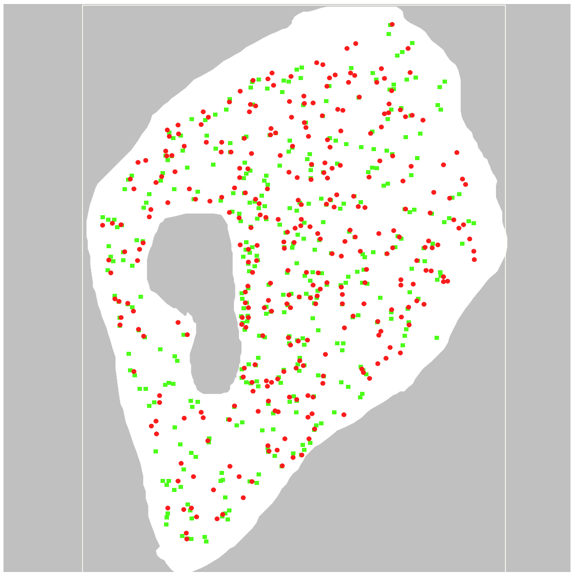

Figure 4: Point pattern with ROI for Lysosomes-GFP/Lysosomes-RFP for the case discussed in the main article.

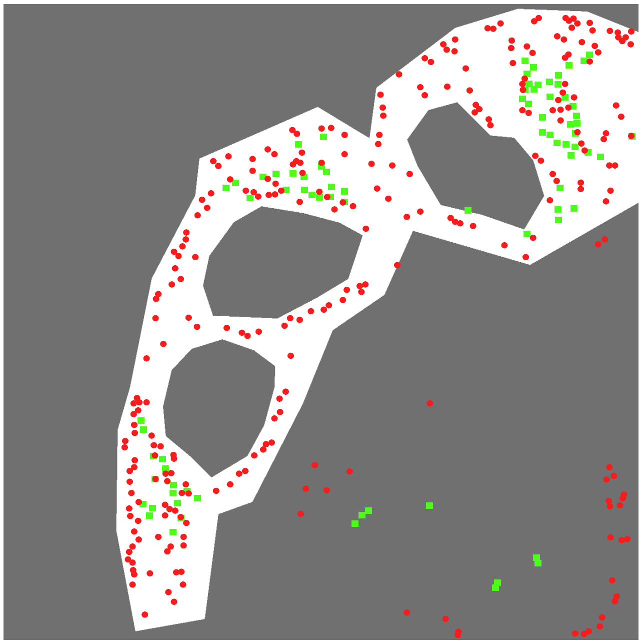

Figure 5: Point pattern with ROI for Golgi-GFP/Lysosomes-RFP for the case discussed in the main article.

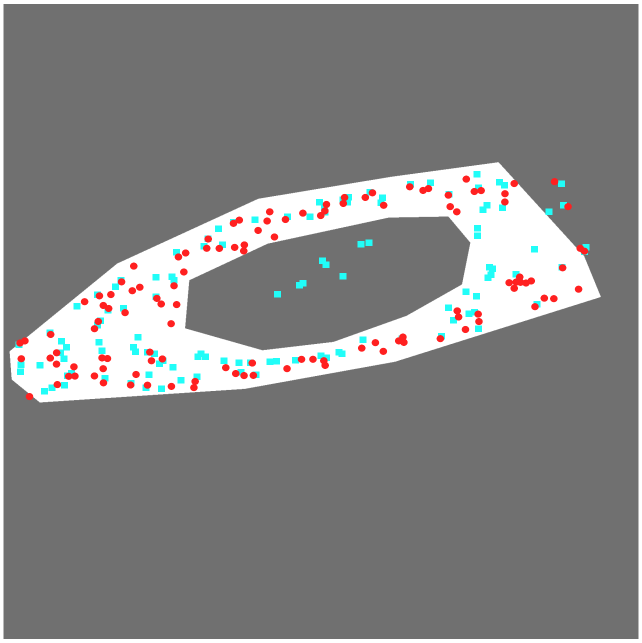

Figure 6: Point pattern with ROI for CND/Lysosomes-RFP for the case discussed in the main article.

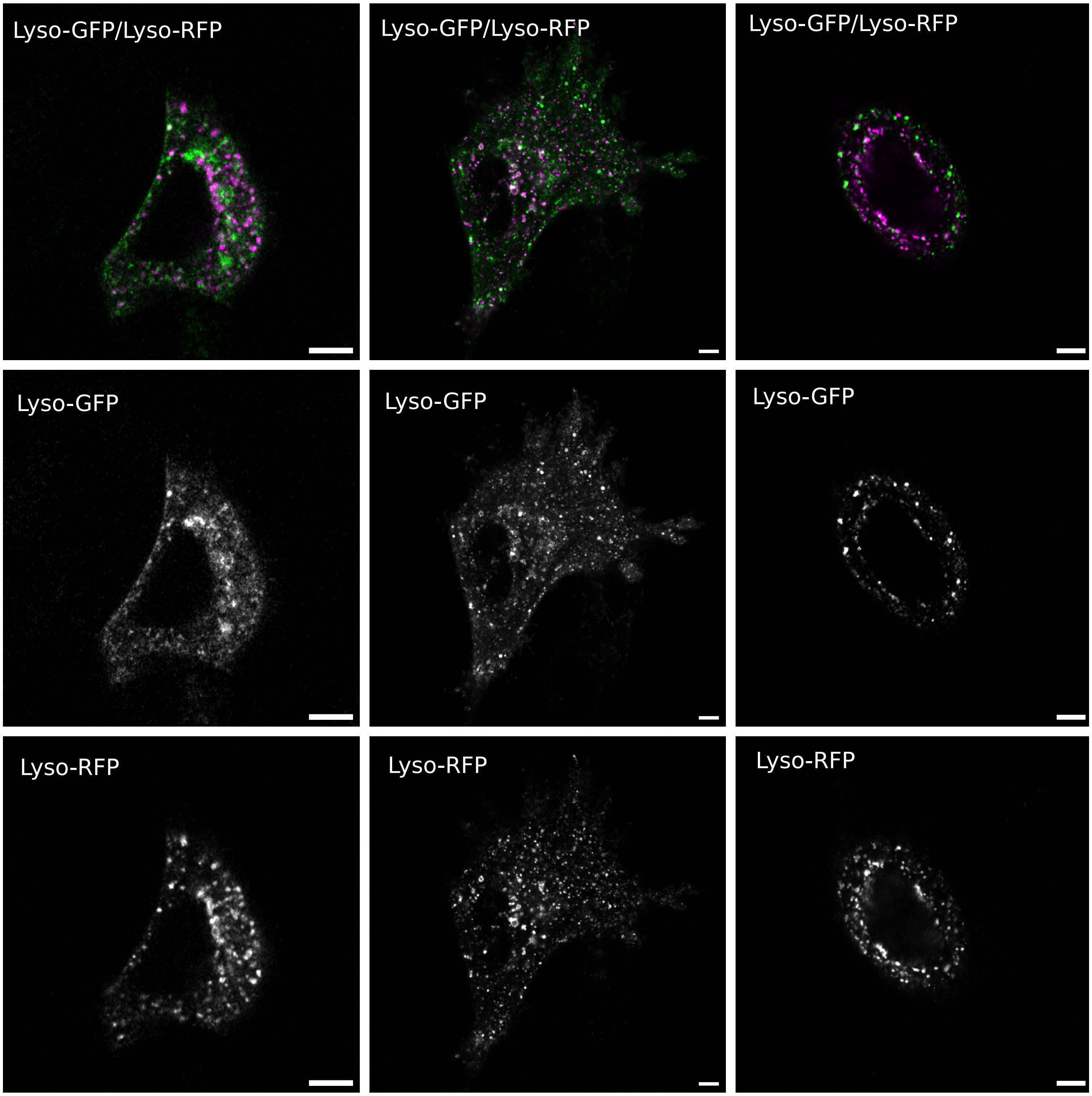

Figure 7: Further microscopy images for Lysosomes-GFP/Lysosomes-RFP. The size of the shown scale bars amounts to $5\,\mu m$.

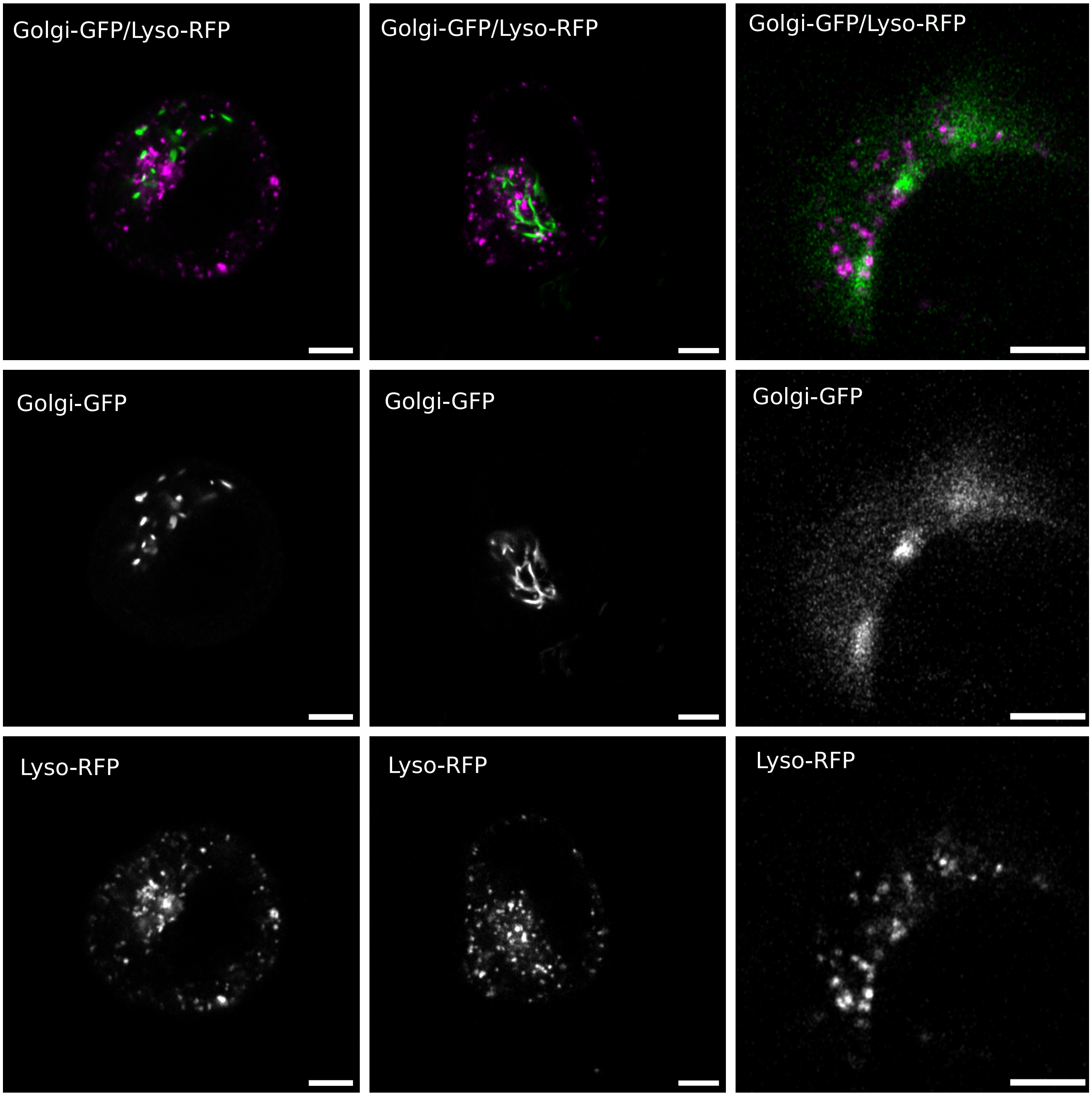

Figure 8: Further microscopy images for Golgi-GFP/Lysosomes-RFP. The size of the shown scale bars amounts to 5 μm.

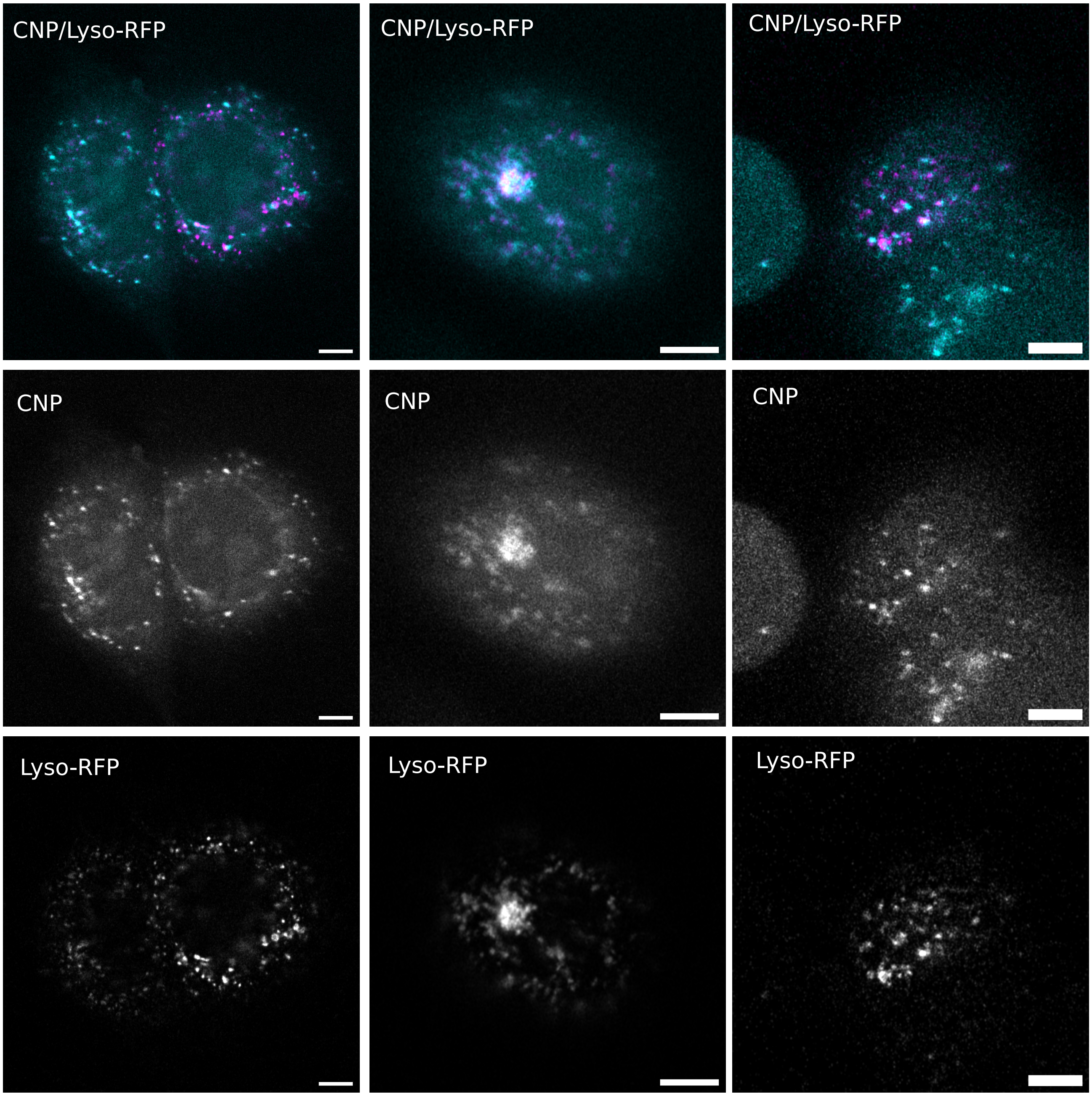

Figure 9: Further microscopy images for CND/Lysosomes-RFPThe size of the shown scale bars amounts to 5 μm.

3 Single Particle Tracking

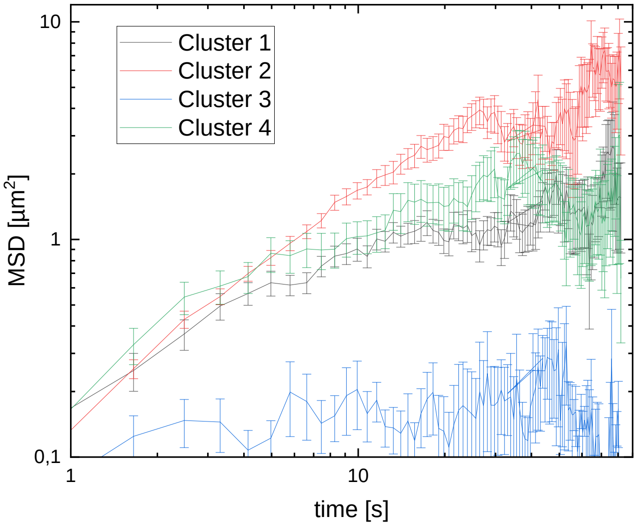

Figure 10: Ensemble averaged mean square displacement plotted against time for all 100 time points.

3.1 Identification of movement patterns via cluster analysis on particle tracking data

3.1.1 Cluster Analysis via tSNE and DBSCAN

To distinguish between different types of motion in the single particle tracking data eight different metrics were calculated. The first four are moments of the distribution of jump distances, namely the mean, the standard deviation, the skewness and the kurtosis of the jump distances in a single track. The four other metrics are global characteristics of the tracks namely the mean squared displacement *msd* [1], the turn angle correlation *tac* [2], the straightness [1] and the sinuosity [3] that were calculated with the *movement_metrics()* function from the *amt-package* [1]. The metrics are given by:

$$msd = \frac{1}{N}\sum_{i=1}^{N}[(x_i-\bar{x})^2+(y_i-\bar{y})^2] \tag{1}$$

$$tac = \frac{1}{N}\sum_{i=1}^{N-1}[(\cos(\alpha_{i+1})-\cos(\alpha_i))^2+(\sin(\alpha_{i+1})-\sin(\alpha_i))^2] \tag{2}$$

$$\text{straightness} = \frac{\text{total distance}}{\text{cumulative distance}} \tag{3}$$

$$\text{sinuosity} = 2\left[p\left(\frac{1-c^2-s^2}{(1-c)^2+s^2}+b^2\right)\right]^{-0.5} \tag{4}$$

With the number of points in a track N, the i-th x- (y-)Koordinate x_i (y_i), the i-th turn angle α_i, the mean step length p, the mean cosine (sine) of α c (s) and the coefficient of variation of step length b. A point in the eight-dimensional space spanned by these metrics is calculated for each track. Prior to the cluster analysis the dimensionality reduction algorithm tSNE [4] is applied mapping the data to two coordinates while preserving the local properties of the eight-dimensional dataset. Clusters are analysed with DBSCAN [5]. For tSNE a perplexity of $perp = 30$ was chosen and for DBSCAN the hyperparameters $\varepsilon = 1.9$ and $minPts = 5$ were chosen. The resulting clusters are displayed in Figure 11. The ensemble averaged mean squared displacement at time t was calculated for each individual cluster (Figure 10).

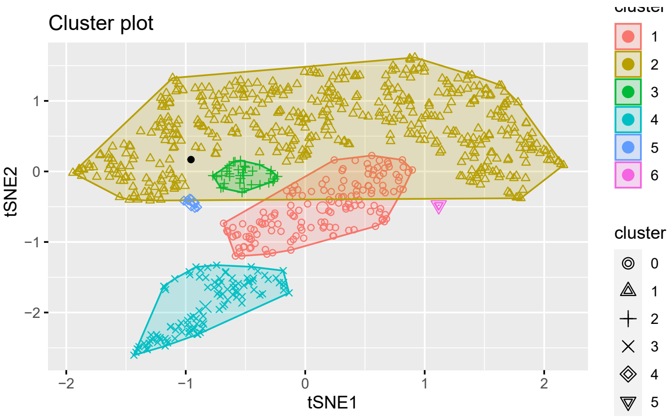

Figure 11: Plot of the DBSCAN clustering on the tSNE-transformed movement metrics for the particle trajectories.

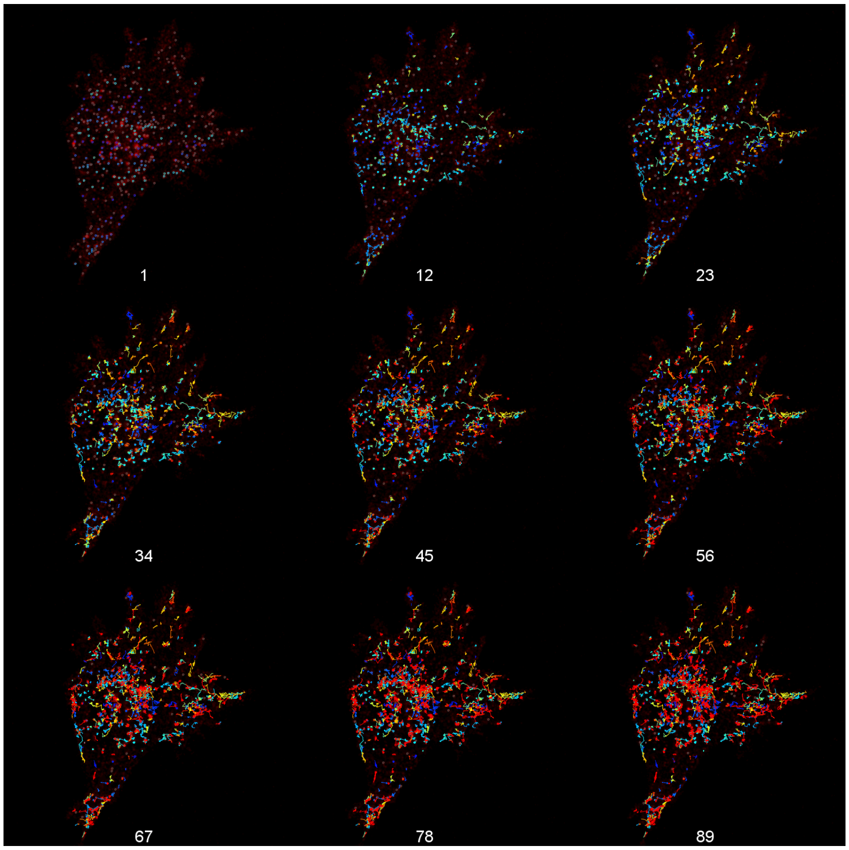

Figure 12: Single particle tracking time series data. The index of the image indicating the position in the time series is displayed in the lower right corner.

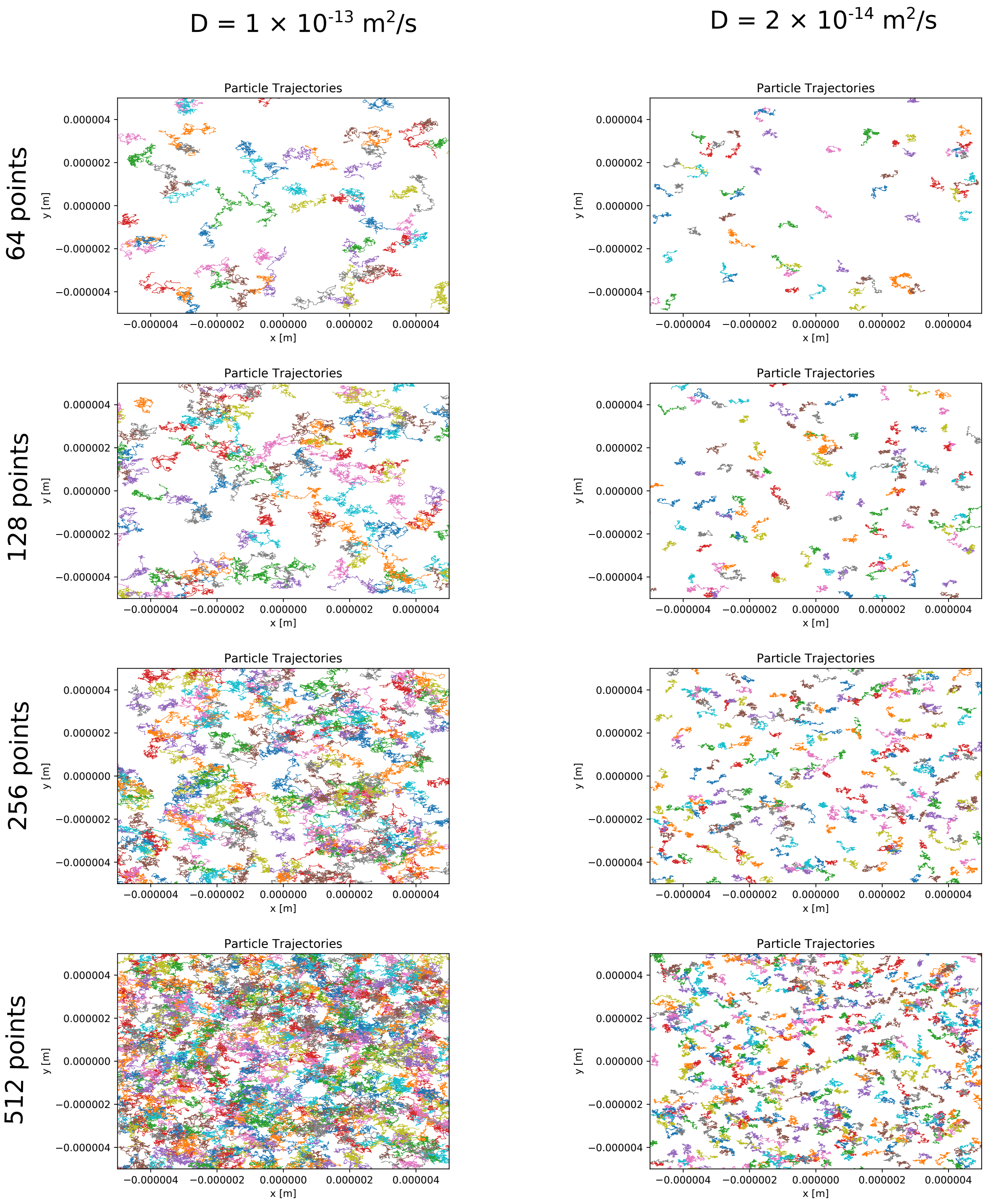

Figure 13: Synthetic data of a diffusion process with a diffusion constant of $D = 1 \times 10^{-13}\,\frac{m^2}{s}$ (left column) and $D = 2 \times 10^{-14}\,\frac{m^2}{s}$ (right column) on a $10\,\mu m \times 10\,\mu m$ region of interest. The positions of the particles were initialized with a Poission point process. The number of particles, and therefore the degree of crowding, here increase from top to bottom.

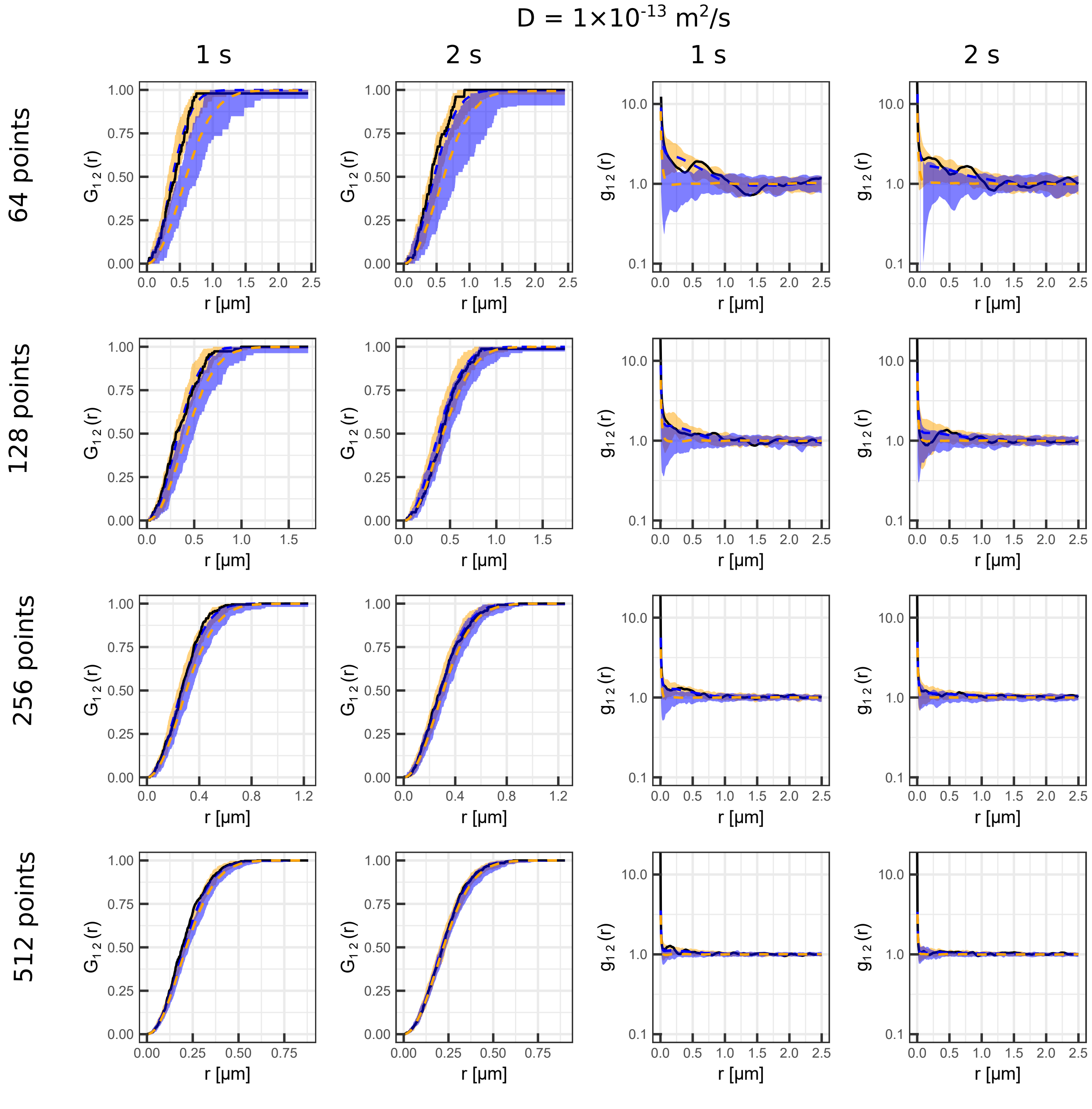

Figure 14: Bivariate pair correlation functions $g_{12}(r)$ and nearest neighbor functions $G_{12}(r)$ (shown as solid black line) calculated from the synthetic data displaying diffusion shown in the left column ($D = 1\,10^{-13}\,\frac{m^2}{s}$) of figure 13 after 1$s$ and 2s correlated with the starting distribution, respectively. The envelope of CSR is displayed in blue, while the envelope of a Monte Carlo simulation of diffusive transport via a 2D Gaussian function is drawn in orange. The number of particles increases from top to bottom illustrating the effect of crowding on the spatial statistics analysis.

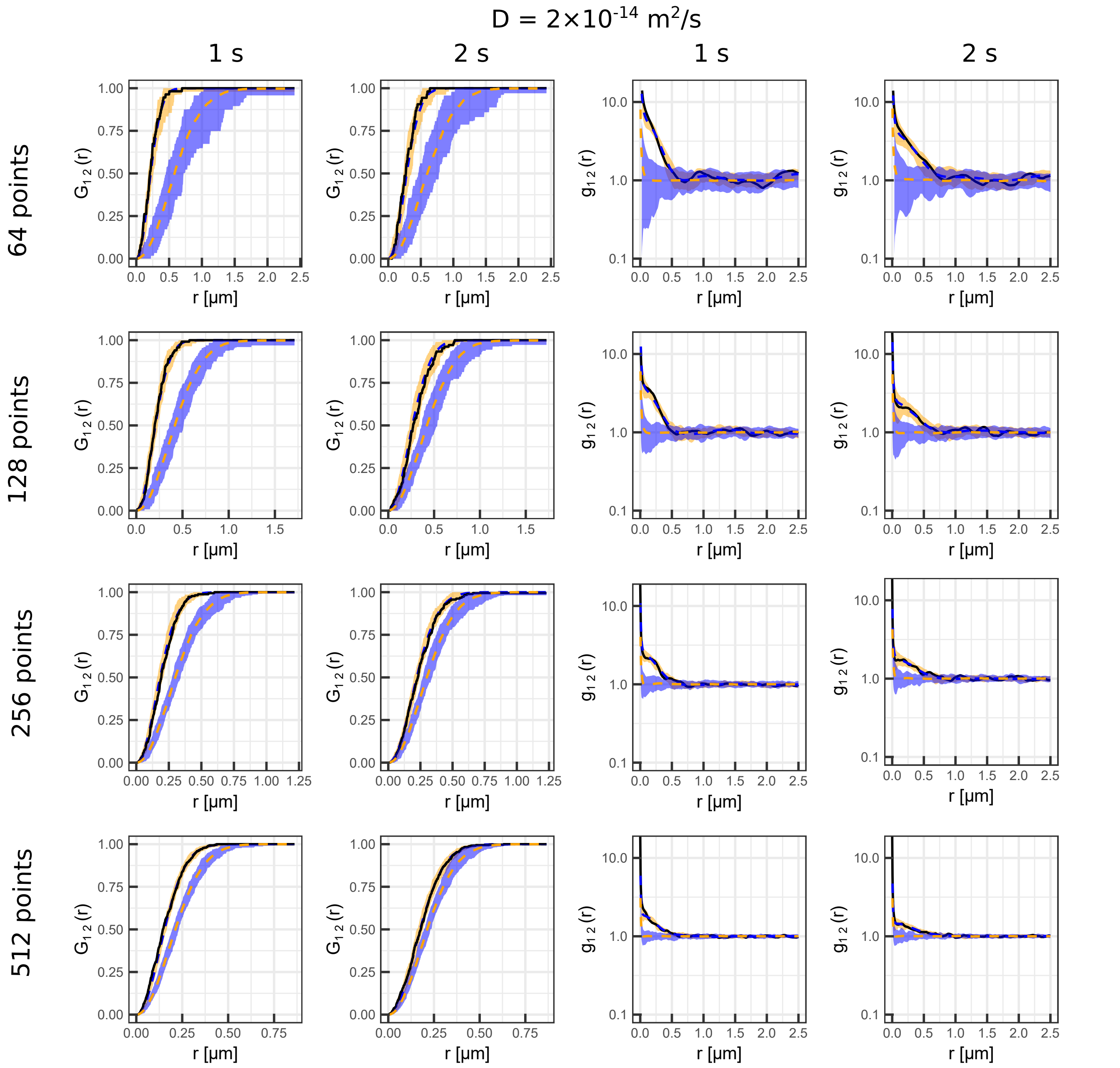

Figure 15: Bivariate pair correlation functions $g_{12}(r)$ and nearest neighbor functions $G_{12}(r)$ (shown as solid black line) calculated from the synthetic data displaying diffusion shown in the right column ($D = 2 \times 10^{-14}\,\frac{m^2}{s}$) of figure 13 after 1$s$ and 2s correlated with the starting distribution, respectively. The envelope of CSR is displayed in blue, while the envelope of a Monte Carlo simulation of diffusive transport via a 2D Gaussian function is drawn in orange. The number of particles increases from top to bottom illustrating the effect of crowding on the spatial statistics analysis.

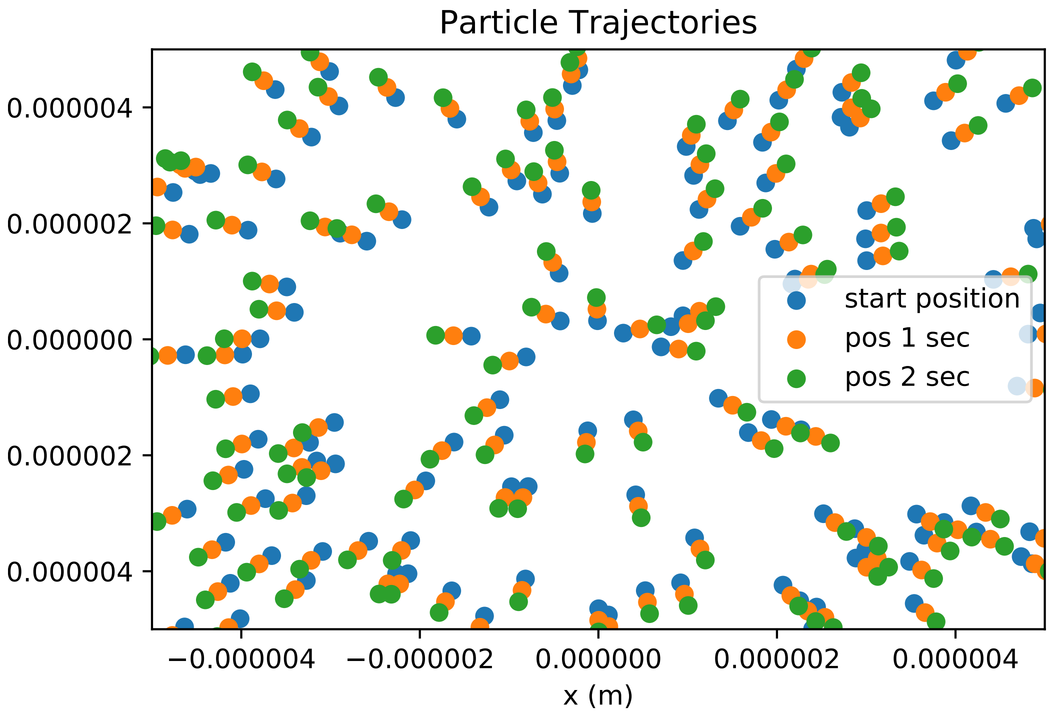

Figure 16: Synthetic bivariate point pattern gernerated by radial drift as an example of a purely directed motion. The point pattern at $t = 0$ is sampled from a random uniform distribution on a $10\,\mu m \times 10\,\mu m$ region of interest. The patterns at $t = 1\,s$ and $t = 2\,s$ are generated by shifting the points in radial direction with a velocity of $2 \times 10^{-9}\,\frac{m}{s}$.

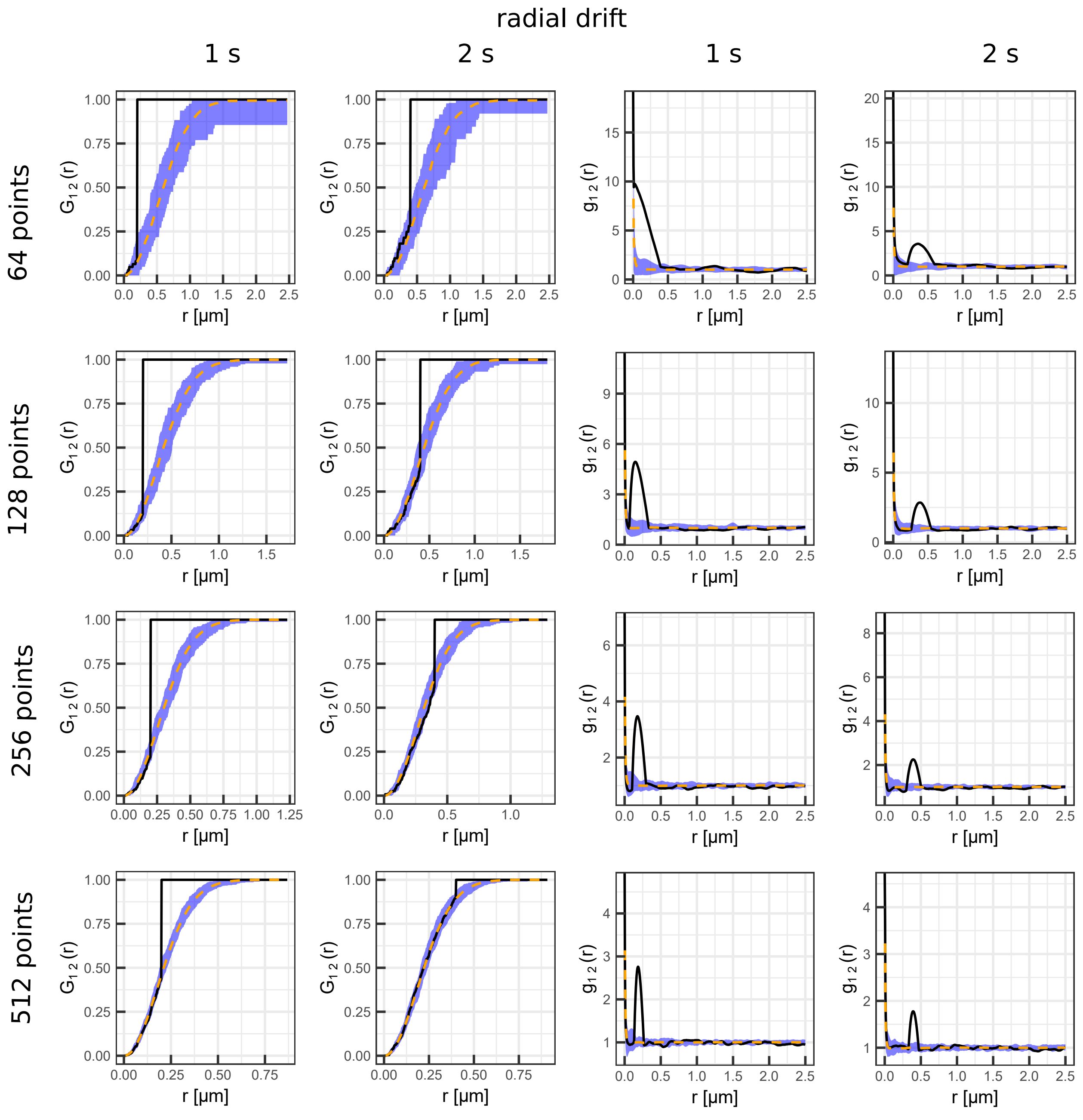

Figure 17: Bivariate pair correlation functions $g_{12}(r)$ and nearest neighbor functions $G_{12}(r)$ (shown as solid black line) calculated from the synthetic data of the point pattern from figure 16, which is subject to a radial drift of a constant velocity of $2 \times 10^{-9} \, \frac{m}{s}$. The summary functions $G_{12}(r)$ and $g_{12}(r)$ were calculated between the starting distribution and the distributions after $t = 1\,s$ and $t = 2\,s$, respectively. The envelope of CSR in channel 2 is shown in blue. The number of particles increases from top to bottom illustrating the effect of crowding on the spatial statistics analysis. Note that $G_{12}(r)$ becomes indistinguishable from CSR with increasing particle density.

References

[1] J. Signer, J. Fieberg and T. Avgar, *Ecology and evolution*, 2019, **9**, 880–890.

[2] B. Abrahms, D. P. Seidel, E. Dougherty, E. L. Hazen, S. J. Bograd, A. M. Wilson, J. Weldon McNutt, D. P. Costa, S. Blake, J. S. Brashares *et al.*, *Movement ecology*, 2017, **5**, 1–11.

[3] P. J. Almeida, M. V. Vieira, M. Kajin, G. Forero-Medina and R. Cerqueira, *Zoologia (Curitiba)*, 2010, **27**, 674–680.

[4] L. Van der Maaten and G. Hinton, *Journal of machine learning research*, 2008, **9**, 2579–2605.

[5] M. Ester, H.-P. Kriegel, J. Sander, X. Xu *et al.*, kdd, 1996, pp. 226–231.
